# Tungsten disulfide-based nanocomposites for photothermal therapy

**DOI:** 10.3762/bjnano.10.81

**Published:** 2019-04-02

**Authors:** Tzuriel Levin, Hagit Sade, Rina Ben-Shabbat Binyamini, Maayan Pour, Iftach Nachman, Jean-Paul Lellouche

**Affiliations:** 1 Institute of Nanotechnology and Advanced Materials & Department of Chemistry, Faculty of Exact Sciences, Bar-Ilan University, Ramat Gan, 5290002, Israel; 2 Department of Biochemistry and Molecular Biology, George S. Wise Faculty of Life Sciences, Tel Aviv University, Tel Aviv, 6997801, Israel

**Keywords:** cerium complex, magnetic nanoparticles, photothermal therapy, surface functionalization, WS_2_ nanotubes

## Abstract

Nanostructures of transition-metal dichalcogenides (TMDC) have raised scientific interest in the last few decades. Tungsten disulfide (WS_2_) nanotubes and nanoparticles are among the most extensively studied members in this group, and are used for, e.g., polymer reinforcement, lubrication and electronic devices. Their biocompatibility and low toxicity make them suitable for medical and biological applications. One potential application is photothermal therapy (PTT), a method for the targeted treatment of cancer, in which a light-responsive material is irradiated with a laser in the near-infrared range. In the current article we present WS_2_ nanotubes functionalized with previously reported ceric ammonium nitrate–maghemite (CAN-mag) nanoparticles, used for PTT. Functionalization of the nanotubes with CAN-mag nanoparticles resulted in a magnetic nanocomposite. When tested in vitro with two types of cancer cells, the functionalized nanotubes showed a better PTT activity compared to non-functionalized nanotubes, as well as reduced aggregation and the ability to add a second-step functionality. This ability is demonstrated here with two polymers grafted onto the nanocomposite surface, and other functionalities could be additional cancer therapy agents for achieving increased therapeutic activity.

## Introduction

In 1992, Prof. Reshef Tenne reported the synthesis of cylindrical and polyhedral nanostructures of tungsten disulfide (WS_2_) [1]. These nanostructures are composed of triple-layer units, where a hexagonal layer of tungsten atoms is sandwiched between two hexagonal sulfur layers. WS_2_ belongs to a family of compounds called transition-metal dichalcogenides (TMDCs), with a general formula of MX_2_ (M = W, Mo and X = S, Se, Te) and a similar structure based on triple-layers.

Good mechanical properties of WS_2_ inorganic nanotubes (INTs; up to 15 µm length, 100 nm diameter) and inorganic fullerene-like nanoparticles (IFs) were reported in multiple literature sources [2–8], making them an excellent alternative to carbon nanotubes as additives for the mechanical enforcement of polymeric matrices [9–17].

An important advantage of WS_2_ (and of other TMDCs) nanostructures over their carbon equivalents is the low toxicity and biocompatibility, enabling their use for medical applications. Preliminary studies on rats with WS_2_ INTs and IFs showed no apparent toxic reaction after oral administration [18], inhalation [19], or dermal application [20]. More recent studies conducted on rhenium-doped MoS_2_ nanoparticles showed no acute toxic risk, neither by oral administration nor by dermal application [21–22]. A few years ago, Teo et al. compared the cytotoxicity of exfoliated MoS_2_, WS_2_, and WSe_2_ to that of their carbon equivalent and found the toxicity of the former to be lower [23]. Wu et al. produced biocompatible MoS_2_ nanoparticles by a pulsed laser ablation technique [24]. Examples of medical applications with TMDC nanostructures are their addition as reinforcing agents to polymers for bone-tissue engineering, and their incorporation in dental devices [25–32]. Another important medical application for nanostructures in general, and for TMDC nanostructures in particular, is targeted cancer treatment through photothermal therapy (PTT). In this method, light-responsive materials accumulate on the tumor area, absorb light, and release it as heat, killing the cancerous cells. The light is generated by a laser, in a near-infrared (NIR) wavelength range (750–1000 nm). NIR irradiation has low off-target interaction and a high penetration depth of ca. 1–2 cm in the human body. PTT mediated by nanomaterials is less invasive than laser irradiation alone, requires lower radiation intensity, and its selectivity towards the tumor can be adjusted by carefully engineering the light-responsive nanostructure. In general, nanomaterials in the size range of 100–200 nm should give the maximum accumulation effect, but parameters such as nanostructure shape and surface charge are extremely important [33].

A wide range of nanomaterials has been studied for cancer PTT to now, from organic conjugated polymers [34–35], through carbon-based nanomaterials [36–37], to inorganic nanostructures. Within the latter group, nanostructures of metals [38], metal oxides (including iron oxides) [39], and metal chalcogenides [40–41] were studied. Tungsten oxides [42] and molybdenum oxides [43] were studied, as well as their disulfides. The disulfides were tested mainly in the forms of nanosheets [44], nanoflakes [45], nanodots [40] and hollow spheres [46]. Recently, WS_2_ nanotubes functionalized with C-dots showed promising results for PTT and cell imaging [47]. We selected nanotubes for their mechanical properties and the possibility of coordinate bonds with sulfur atoms, which enables bonding with CAN-mag, thereby offering the possibility of bonding of additional biologically active agents. The properties of the CAN-mag also enabled magnetism-based targeting.

In order to maximize the benefit from TMDC nanostructures in different applications, their surface functionalization is important. The relative chemical inertness of the outer chalcogen layer makes TMDC nanostructures very hard to disperse in many solvents, especially in water. This is a significant limitation when attempting to use these nanostructures for biological applications. Functionalization, especially the attachment of organic moieties to the walls of TMDC nanostructures is a challenging task. Coordinative chemistry is one approach used to face this challenge.

The Tremel group has been successfully developing coordination-chemistry-based strategies for the functionalization of TMDC nanostructures for more than a decade. In 2006, they reported a method based on a nickel–nitrotriacetic acid (Ni-NTA) complex as an anchoring group for different chemical functionalities [48–50]. Another study of the Tremel group involves terpyridine (TerPy) ligands [51]. NTA and TerPy are multi-dentate ligands, forming complexes with chalcophylic metal ions (nickel, iron, ruthenium) and leaving parts of the ion coordination sites free for docking to the chalcogen layer. The molecular structure of both NTA and TerPy enables performing versatile chemistry on the ligand.

Cerium is a metal of the lanthanide series with versatile coordinative chemistry, thanks to an available valence electron in its 4f orbital. In our group, cerium was utilized in the complex form of ceric ammonium nitrate [(NH_4_)_2_Ce(IV)(NO_3_)_6_, or CAN]. In CAN the cerium ion is coordinated with six nitrate ligands through their oxygen atoms. CAN is a strong oxidizer, turning magnetite nanoparticles into γ-maghemite (mag) nanoparticles. The cerium ion attaches to the nanoparticle, producing surface defects (an Fe–O–[CeL*_n_*] bond is formed). The cerium-doped maghemite nanoparticles are more stable than the non-doped ones, which tend to aggregate. In addition to the stabilization effect, other ligands on the cerium ion can be replaced by different polymers and linkers. The resulting nanocomposites can be used for biomedical applications, such as gene silencing [52], magnetic imaging, and drug delivery.

Here we present a new and simple-to-fabricate WS_2_-NT-CAN-mag (WS_2_-NT-CM) nanocomposite. The composite is magnetic and forms a stable dispersion in water. It was characterized to verify the CAN-mag attachment to the nanotubes, and tested for PTT. Preliminary in-vitro PTT tests show that the WS_2_-CAN-mag nanocomposite successfully eliminated two types of cancerous cells: HeLa cells (cervical cancer) and MCF7 cells (breast cancer), in higher percentages compared to non-functionalized nanotubes. In addition, WS_2_-CAN-mag nanocomposites with two types of organic polymers were successfully prepared and characterized. Indeed, functionalization with CAN-mag gives WS_2_ nanotubes the added values of reduced aggregation, which leads to better targeting, and the possibility for attachments of additional cancer therapy agents.

## Experimental

**Figure 1:**
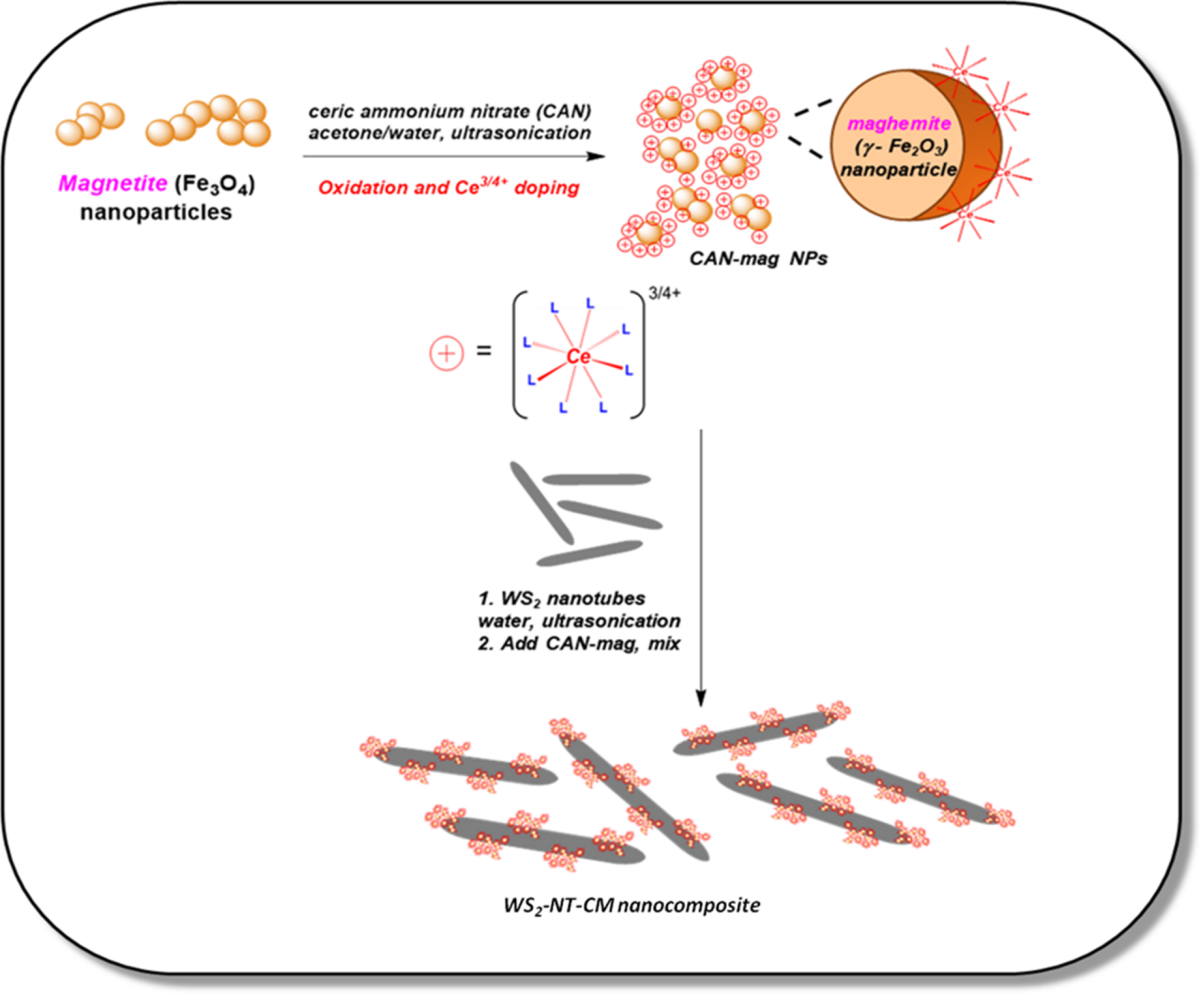
Schematic description for WS_2_-NT-CM nanocomposite preparation.

A schematic description of the experimental pathway leading to CAN-mag functionalized WS_2_ nanotubes is given in Figure 1, followed by fully detailed preparation procedures.

### Preparation of CAN-mag nanoparticles

A solution of FeCl_3_·H_2_O (240.0 mg, 0.9 mmol) in degassed, ddH_2_O water (4.5 mL) was mixed with an aqueous solution of FeCl_2_·4H_2_O (97.5 mg, 0.45 mmol, 4.5 mL H_2_O). The mixture was kept under nitrogen and ultra-sonicated for 1 min at room temperature. Then, a concentrated (24 wt %) NH_4_OH solution (750 µL) was added, resulting in the immediate formation of a black precipitate of magnetite (Fe_3_O_4_) nanoparticles. Sonication was continued for an additional 10 min. The liquid was decanted with the help of magnetic separation, using a 0.5 T magnet. The nanoparticles were washed with three portions of ddH_2_O (40 mL each) to neutrality. Then, ddH_2_O (30 mL) was added, and the maghemite NPs suspension was set aside for a minimum of 1.5 h at ambient temperature for aging, before any further use.

A solution of CAN (500.0 mg, 0.912 mmol) in acetone (6.0 mL) was added to the decanted magnetite NPs, followed by the addition of degassed purified water (18 mL). The resulting mixture was ultrasonicated for 30 min under nitrogen using a high-power sonicator, then transferred into 50 mL Amicon^®^ Ultra-15 centrifugal filter tubes (100KD, Millipore, Cork, Ireland). The contents were washed with three portions of ddH_2_O (10 mL each), and centrifuged at 4000 rpm for 10 min at 18 °C each time. The washed nanocomposite was dispersed in ddH_2_O (25 mL). The iron concentration in the dispersion was determined by the inductively coupled plasma (ICP) method to be 2.7 mg/mL.

### Preparation of WS_2_-NT-CM nanocomposite

WS_2_-NTs (15 mg, NanoMaterials Ltd., Yavne, Israel; Lot number: TWPO-MA018) were dispersed in ddH_2_O (15 mL) using an ultrasonic probe (set to reach 3.7 KJ, with 20% amplitude) for 7 min at room temperature. Then, the aqueous CAN-mag dispersion (550 µL) was added (this volume gives a 1:10 weight ratio between the iron and the WS_2_-INTs). The mixture was shaken for 24 h at ambient temperature. WS_2_-NT-CM was separated from the solution using a 0.5 T magnet, washed with three portions of ddH_2_O (20 mL each, no centrifugation), and dried by using a lyophilizer.

### Preparation of polymer-functionalized WS_2_-NT-CM nanocomposites

WS_2_-NT-CM (20 mg) was dispersed in ddH_2_O (75 mL) using an ultrasonic bath. Then, polyethylenimine (11.5 mg, branched PEI, *M*_w_ ≈ 25000, Sigma-Aldrich, St. Louis, MO, US) dissolved in ddH_2_O (1 mL) was added. The mixture was shaken at 15 °C for 48 h (220 RPM). The WS_2_-NT-CM-PEI was washed with 3 portions ddH_2_O (50 mL each, 5000 RPM, 5 °C, 10 min) and dried by using a lyophilizer. Alternatively, polyacrylic acid (25 mg, PAA, sodium salt, *M*_w_ ≈ 8000, 45% aq. Sol., Sigma-Aldrich, St. Louis, MO, US) dissolved in ddH_2_O (1 mL) was added, and the mixture was shaken at 10 °C for 72 h.

### Characterizations

ATR-FTIR spectra were obtained on a Nicolet iS5 FT-IR spectrometer (Thermo Scientific, Waltham, MA, US) equipped with an iD5 ATR accessory featuring a laminated diamond crystal. Samples were analyzed without further preparation. The data processing was performed using OMNIC 9 spectra software (Thermo Scientific, Waltham, MA, US).

Thermogravimetric analysis (TGA) was performed by employing a TGA/DSC1 analyzer (Mettler-Toledo, Greifensee, Switzerland). All thermograms were recorded in a nitrogen (50 mL/min) environment at a heating rate of 10 °C·min^−1^ over the temperature range of 30–800 °C. Weight change and heat flow were measured simultaneously during the analysis. The results were processed using STARe evaluation software (Mettler-Toledo, Greifensee, Switzerland).

Transmission electron microscopy (TEM) images were acquired by a Tecnai Spirit Bio-Twin microscope (FEI, Hillsboro, OR, US) equipped with a 1k × 1k CCD camera (Gatan, Pleasanton, CA, US). Samples for TEM analysis were dispersed in water. A drop of the dispersion was placed on a formvar/carbon film on a 400-mesh copper TEM grid (FCF400-Cu, Electron Microscopy Sciences, Hatfield, PA, US) and then dried at ambient temperature for 24 h.

High-resolution transmission electron microscopy (HRTEM) images were acquired using a high-resolution transmission electron microscope (JEM 2100, JEOL Inc., Peabody, MA, US) equipped with a 4k × 4k CCD camera (Gatan, Pleasanton, CA, US). Samples were prepared using the same procedure as for TEM analysis.

High-resolution scanning electron microscopy (HRSEM) images were acquired using a Magellan 400L high-resolution scanning electron microscope (FEI). Samples for HRSEM were prepared by placing a few drops of the aqueous dispersion of the dried sample on a square piece of a clean silicon wafer and drying overnight at ambient temperature.

Zeta potential measurements were performed using a Zetasizer Nano-ZS device (Malvern Instruments Ltd., Worcestershire, UK). Samples for zeta potential measurements were dispersed in water (ca. 0.5 mg/mL).

Inductively coupled plasma (ICP) was used to determine the concentration of cerium and iron (Ultima-2 instrument, Horiba [Jobin-Yvon division], Kyoto, Japan). For cerium analysis, lyophilized nanocomposite sample (2–5 mg) was dissolved in concentrated hydrochloric acid (350 µL, DaeJung, Busan, Korea), diluted to 10 mL with dd water, and set aside overnight for decomposition. The solution was then filtered through a 0.22 µm PTFE syringe filter (Millipore, Darmstadt, Germany). For iron analysis, 1 mL of the filtered solution was diluted to 10 mL with dd water.

Superconducting quantum interference device (SQUID) measurements were performed (MPMS-5XL magnetometer, Quantum Design, San Diego, CA, US). For analysis, dried samples were placed in a plastic capsule. The measurements were run at a temperature of 100 K.

### Photothermal therapy activity

For photothermal therapy experiments, we tested two different human cancer cells – HeLa (cervical cancer) and MCF7 (breast cancer). The cells were cultured on 24-well plates. When the cells reached 80% confluence, freshly prepared aqueous dispersions of WS_2_-NT or WS_2_-NT-CM (45 µL, 1 mg/mL) were added to two of the plates, and a third plate, with no additives, was used for control. After 10 min of incubation, the cells were washed three times with PBS buffer and a fresh DMEM medium was added. For each condition, four representative frames were imaged under a Zeiss LSM7 inverted two-photon microscope at 10× magnification in phase-contrast. Next, a square region of 157 µm × 157 µm in the middle of each frame was irradiated with a 700 nm laser (Chameleon Vision II) at 123 mW for 1 min. The same frames were then imaged again. A dye exclusion test of cell viability was performed, using Trypan Blue for staining. A mixture of 0.5 wt % trypan blue solution and PBS (1:1 v/v) was added to all wells after the laser irradiation. After 5 min, the cells were washed with PBS buffer and the same frames were imaged.

## Results and Discussion

**Figure 2:**
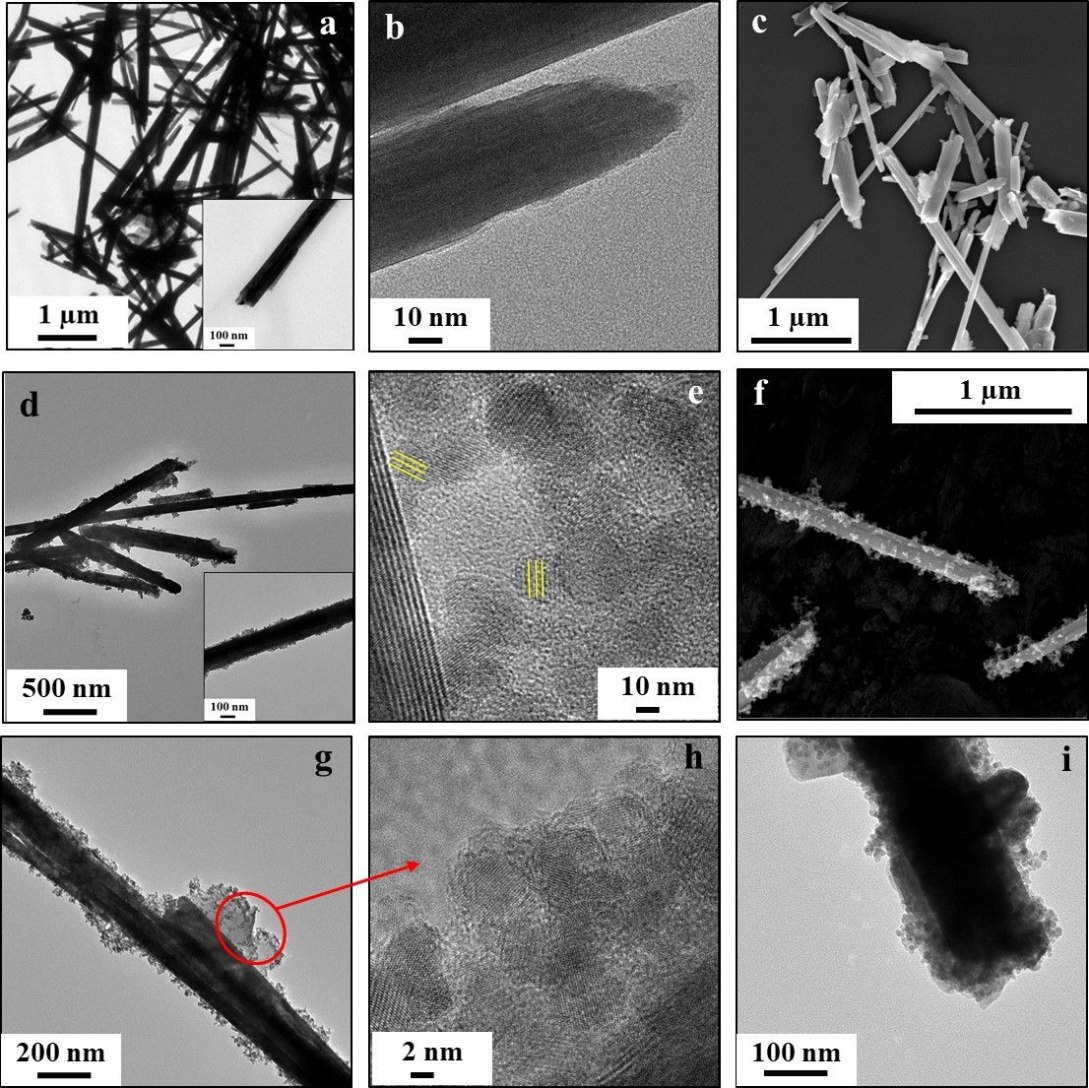
TEM, HRTEM, and SEM images of (a–c) WS_2_-NT; (d–f) WS_2_-NT-CM; (g,h) WS_2_-NT-CM-PEI and (i) WS_2_-NT-CM-PAA.

This section will include all the chemical and biological results. The preparation procedures of the composites included sonication steps that might cause massive breakage or exfoliation of the nanotubes. For this reason, we aimed for preparation conditions that would allow composite formation without damaging the nanotubes. Electron microscopy images (Figure 2) show that WS_2_ nanotubes maintained their general shape after conjugation of CAN-mag nanoparticles, and later on, of the polymers. At the same time, attachment of CAN-mag to the nanotubes is clearly visible (Figure 2d–f). Rather than conformally coating the nanotubes, CAN-mag nanoparticles seem to attach to the surface of the nanotubes in small clusters, appearing dark in TEM, and bright in SEM. A possible reason for this is that CAN-mag composite has a strong positive surface charge (see zeta potential results below in Figure 7), causing electrostatic repulsion forces that prevent a denser coverage. Another point that the electron microscope images show (see Figure 2a and Figure 2c; cf. Figure 2d and Figure 2f), is that WS_2_-NT-CM is significantly less aggregated in aqueous dispersion compared to WS_2_-NT. Here, too, the electrostatic repulsion provided by CAN-mag is probably the reason. In the HRTEM image of WS_2_-NT-CM (Figure 2e), the crystalline nanoparticles of maghemite are easily observed, including visible lattice fringes (marked in yellow). TEM images of WS_2_-NT-CM-PEI (Figure 2g) and WS_2_-NT-CM-PAA (Figure 2i) show that the dark CAN-mag composite is surrounded by a lighter substance, namely the organic polymer (PEI or PAA). A closer look by HRTEM into WS_2_-NT-CM-PEI (Figure 2h) shows that the wavy-looking matrix surrounding the crystalline maghemite is amorphous.

Table 1 shows the results of ICP analysis of CAN-mag nanoparticles and WS_2_-NT-CM composite. The ratios between iron and cerium are very close when comparing the nanoparticles and the composites. This means that there was almost no detachment of CAN during the composite preparation, which is a possibility when using probe sonication. The numbers show a small quantity of cerium in CAN-mag, which is even smaller within the composite, yet the presence of cerium still allows coordinative attachment of polymers to the composite.

**Table 1:** ICP results for CAN-maghemite nanoparticles before and after conjugation to WS_2_ nanotubes. CAN/maghemite molar ratio calculation is based on two moles of iron in each mole of maghemite.

	element in composite [wt %]		Fe/Ce	mag/CAN
	iron	cerium	weight ratio	molar ratio

CAN-mag NPs	71.1 ± 0.1	1.84 ± 0.01	39	48
WS_2_-NT-CM	8.6 ± 0.1	0.21 ± 0.01	41	51

**Figure 3:**
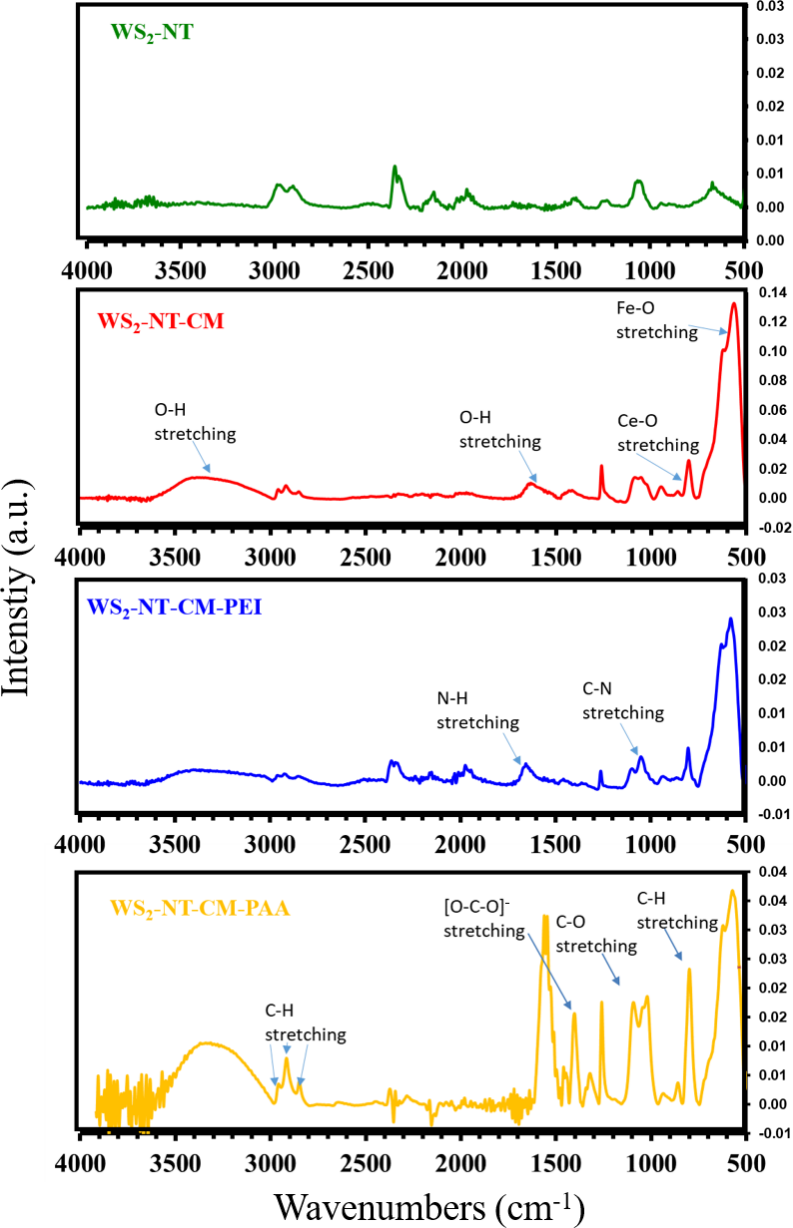
FTIR absorbance spectra of WS_2_-NT and its nanocomposite samples.

Figure 3 shows the FTIR absorbance spectra of WS_2_-NT and its composites. The absorption of the WS_2_-NTs was so weak that we are not sure that anything can be learned from such absorption. In the spectrum of WS_2_-NT-CM (and of its two composites), the strong peak at 570 cm^−1^ is characteristic of iron oxides, and represents the stretching vibration of Fe–O bond [53–54]. The peaks at 1640 cm^−1^ and 3400 cm^−1^ originate from interlayer water: the former is assigned H–O–H bending vibrations, and the latter to O–H stretching vibrations [53–54]. The peak at around 820 cm^−1^ might be assigned to Ce–O stretching vibrations [55]. In the spectrum of WS_2_-NT-CM-PEI, the peaks at 1050 cm^−1^ and 1640 cm^–1^ are a bit more accentuated compared to the WS_2_-NT-CM spectrum. These bands may be assigned to the C–N stretching vibrations and N–H stretching vibrations, respectively, of the PEI chains. The peak at 2350 cm^−1^ is typical to CO_2_, most likely captured by PEI [56]. In the spectrum of WS_2_-NT-CM-PAA, the peaks originating from the polyacrylic acid are dominant. The peak at 800 cm^−1^ is assigned to C–H bending vibrations in the PAA chain. The peaks in the range of 1000–1260 cm^−1^ may be assigned to C–O stretching vibrations. The peaks at around 1400 cm^−1^ and 1540 cm^−1^ are assigned to symmetric and asymmetric stretching vibrations of carboxylate [O–C–O]^−^ ions. The positions of the peaks indicate attachment of the polymer ligand to the surface of the maghemite nanoparticles through carboxylate groups. In the species –COO–Fe, these are shifted to slightly higher energies compared to non-attached carboxylates [57–58]. The peaks at 2850 cm^−1^, 2920 cm^−1^, and 2960 cm^−1^ are assigned to C–H stretching vibrations in the PAA chain. The broad peak at 3400 cm^−1^ is stronger compared to the other spectra. PAA is a very hygroscopic polymer, and the absorbed water contributes to the intensity of the OH band.

**Figure 4:**
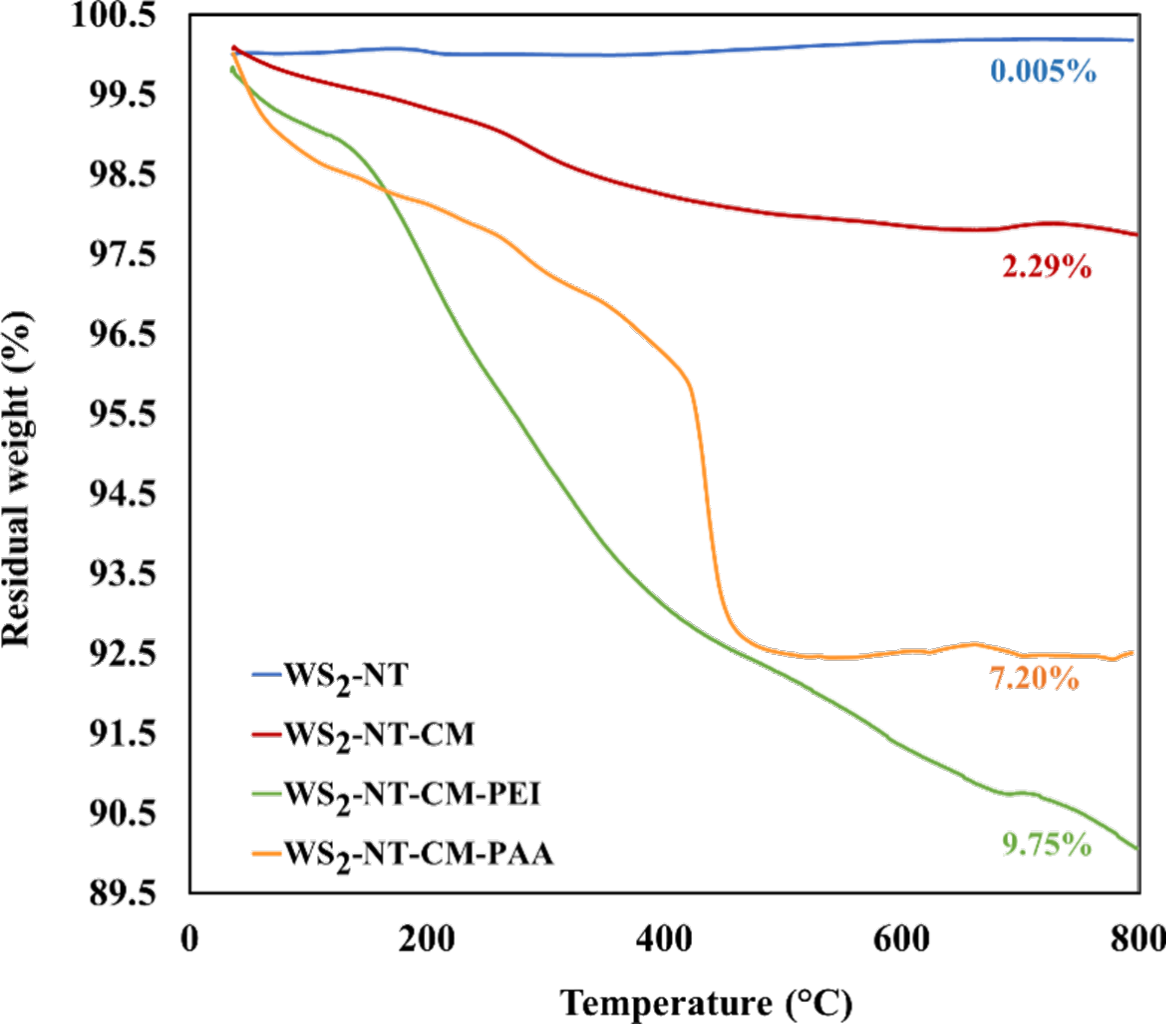
TGA analysis data of WS_2_-NTs and its nanocomposites. The analysis was conducted under nitrogen. Weight loss percentages appear near each thermogram.

Figure 4 shows the thermogravimetric analysis (conducted under nitrogen) results for WS_2_-NT and its composites. WS_2_-NTs (blue line) show almost no weight loss, as expected under these conditions. The nanotubes with CAN-mag functionalization (red line) show a small and gradual weight loss, at a relatively low temperature range, assigned to the organic ammonium and nitrate components of CAN (cerium and iron oxide are not expected to be affected under nitrogen). WS_2_-NT-CM-PAA (yellow line) starts with a relatively steep weight loss step, most probably due to adsorbed water molecules (as mentioned, PAA is highly hygroscopic). A more significant weight loss reaches its plateau around 500 °C, typical for PAA [59–60]. For WS_2_-NT-CM-PEI (green line), there seem to be two sequential weight-loss steps overlapping at approximately 400 °C. The first and major one is typical to PEI [61–62], and the second one, at higher temperatures, originates from a mixed PEI-organic matter polyCOOH/[Ce^3/4+^L*_n_*] complex adlayer phase [63].

**Figure 5:**
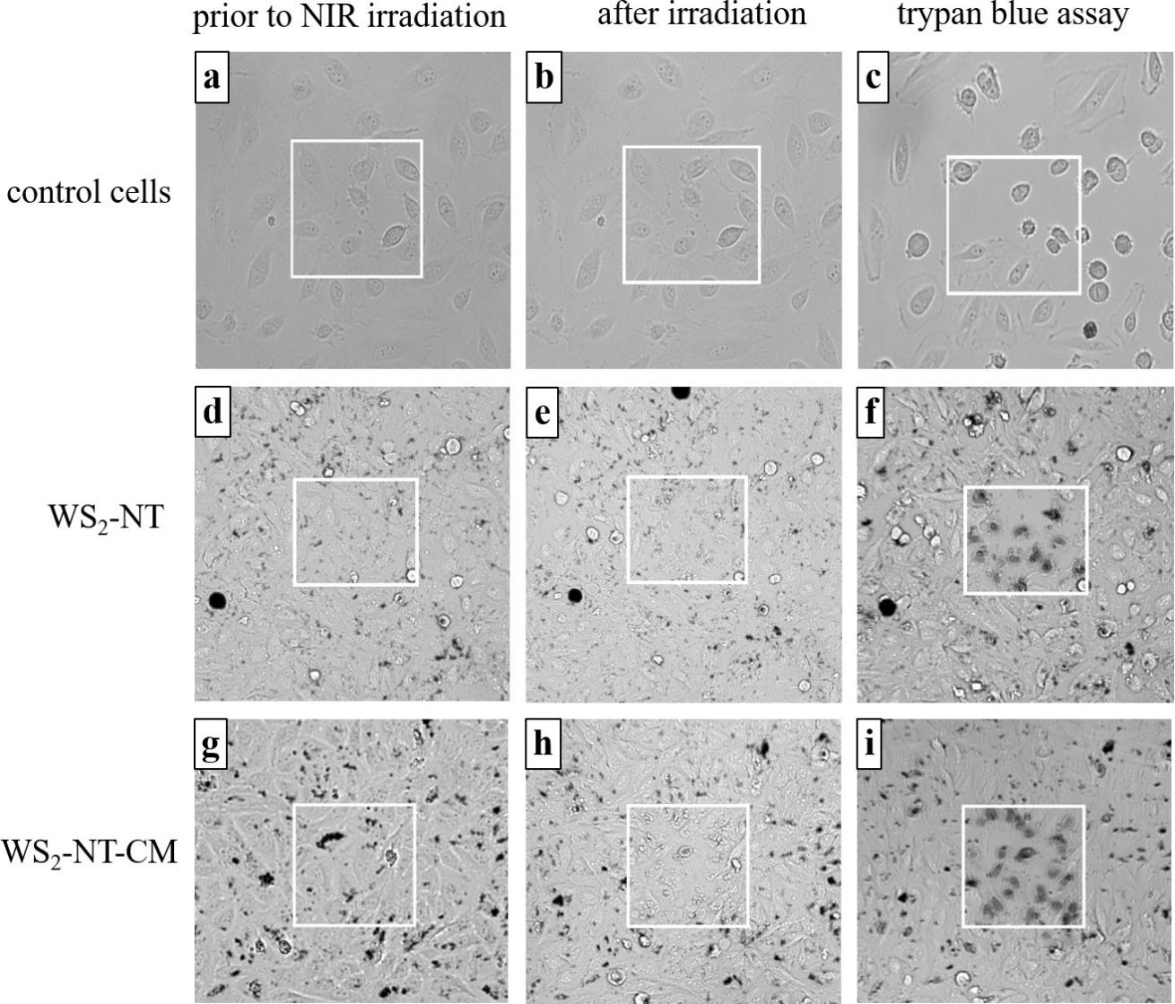
Phase-contrast microscopy images of HeLa cells. The white squares indicate 157 µm × 157 µm areas irradiated with an NIR (700 nm) laser. Left column: cells prior to NIR irradiation; middle column: after irradiation for 1 minute; right column: after irradiation for 1 minute and application of trypan blue assay: (a–c) control (untreated) cells; (d–f) cells pre-incubated with WS_2_-NT; (g–i) cells pre-incubated with WS_2_-NT-CM.

**Figure 6:**
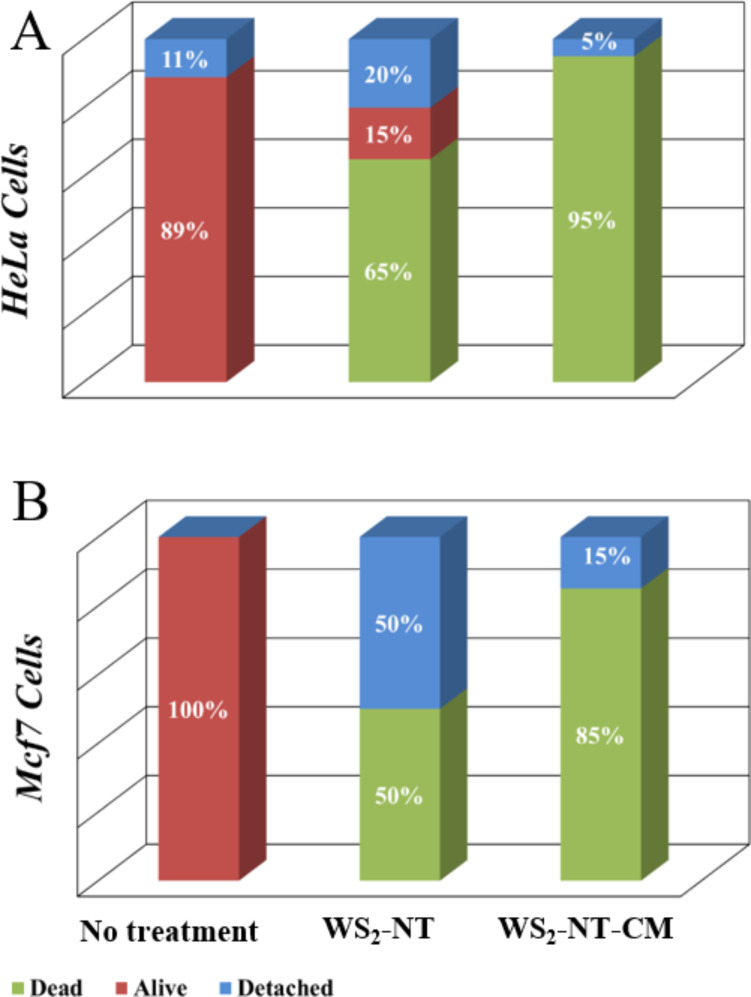
PTT results histograms for WS_2_ nanotubes and WS_2_-NT-CM nanocomposite, tested with HeLa(A) and MCF7(B) cancer cells. Percentages of dead, alive and detached cells are shown for each test.

Figure 5 and Figure 6 demonstrate the application of WS_2_-NT-CM composite as a photothermal therapy agent. Figure 5 shows optical microscope images taken from a cell viability test of HeLa cells incubated for 10 min with WS_2_-NTs (d–f), with WS_2_-NT-CM (g–i), and without any addition (a–c) for reference. Figure 6 shows the percentage of alive, dead, and detached HeLa (A) cells and MCF7 (B) cells after incubation and irradiation (percentages are averaged from three repetitions for each viability test; for the images of the viability test with MCF7 cells, see Supporting Information File 1). The incubated cells were irradiated with a 700 nm NIR laser for 1 min. The irradiated area in each image within Figure 5 is represented by a white square. In the left column are the incubated cells before irradiation, in the middle column after irradiation, and in the right column after irradiation and application of trypan blue. Notice that the entire area in the images was stained, but only the squared area was irradiated. Only dead cells are dyed by trypan blue, and in the images they appear gray and blurry due to the collapse of the cell membrane and the penetration of the dye. The images show cell death only in the squared area, for only the cells incubated with the nanomaterials. This means that the cell death was not caused by irradiation alone or by the addition of the nanomaterials alone, but by the combination of both, proving a photothermal activity. Looking at Figure 5f and Figure 5i, cell death is observed both when incubated with bare WS_2_-NTs and with WS_2_-NT-CM. However, cell death is more accentuated after addition of the latter (Figure 5i). This is also expressed in higher percentages of dead HeLa cells. For MCF7 cells, the cell viability results are less conclusive compared to HeLa cell results (see Supporting Information File 1). While a comparison between Figure S2f and Figure S2i shows more dead cells in the latter, half of the cells tested with WS_2_-NTs were detached during the viability test. Those cells were most likely dead as well, meaning that the advantage of WS_2_-NT-CM over WS_2_-NT in the elimination of MCF7 cells is not distinct.

There is another advantage of the composites over their components alone. WS_2_-NTs tend to aggregate, and the addition of CAN-mag reduces aggregation. Aqueous dispersions of the functionalized nanotubes stay stable for hours, while bare nanotubes in water sink almost instantly. Also, when comparing the images in the bottom and middle rows for both cell types, we see that the WS_2_-NT-CM composite tends to preferably accumulate in and near the cells, while the bare WS_2_-NTs are distributed all around in the imaged area, at times in large aggregates. For targeted PTT purposes, the use of bare nanotubes is not practical, because they will aggregate on the walls of the blood vessels and not reach the tumor area. The use of CAN-mag alone, on the other hand, is not good either, as it will undergo filtration by the liver [64]. So overall, there is a double advantage of WS_2_-NTs functionalized with CAN-Mag, namely increased cancerous-cell death and better targeting.

**Figure 7:**
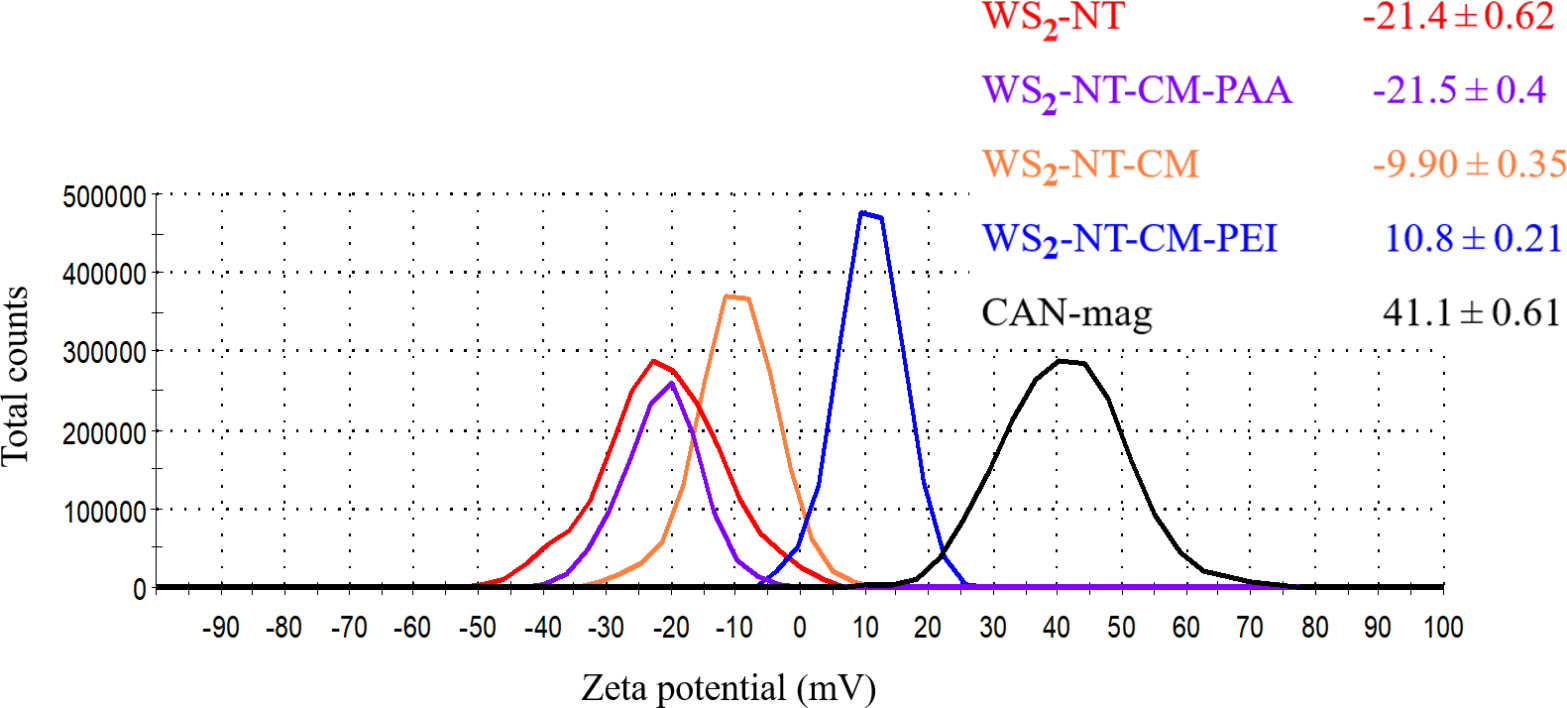
Zeta potential values and distribution curves for samples WS_2_-NT-CM-PEI (blue), WS_2_-NT (red), WS_2_-NT-CM (Orange), WS_2_-NT-CM-PAA (purple), and CAN-mag (Black). For each sample, three measurements were taken, and the values given include the average result and the standard deviation.

Figure 7 shows zeta potential averages and distribution curves for WS_2_-NTs, CAN-mag, and their composites. The values for WS_2_-NT and CAN-mag are consistent with previous works [52,65–66]. For each composite, the zeta values reflect the contributions of the components. The presence of the positively charged CAN-mag on the surface of WS_2_-NTs shifts their value from −21.4 mV to −9.90 mV. The fact that the zeta potential value of WS_2_-NT-CM is not positive is another indication of the inhomogeneity of the coating, as seen in the TEM and SEM images (Figure 2). The addition of polymers to WS_2_-NT-CM has a stronger influence on the zeta potential of the resulting composites. The value shifts more towards the zeta potential of the polymer, because the polymer constitutes the surface. PEI is highly positively charged in water because of the many protonated amine groups, and the WS_2_-NT-CM-PEI composite becomes positively charged. PAA is negatively charged (carboxylate groups), shifting the composite from −9.90 mV to −21.4 mV.

**Figure 8:**
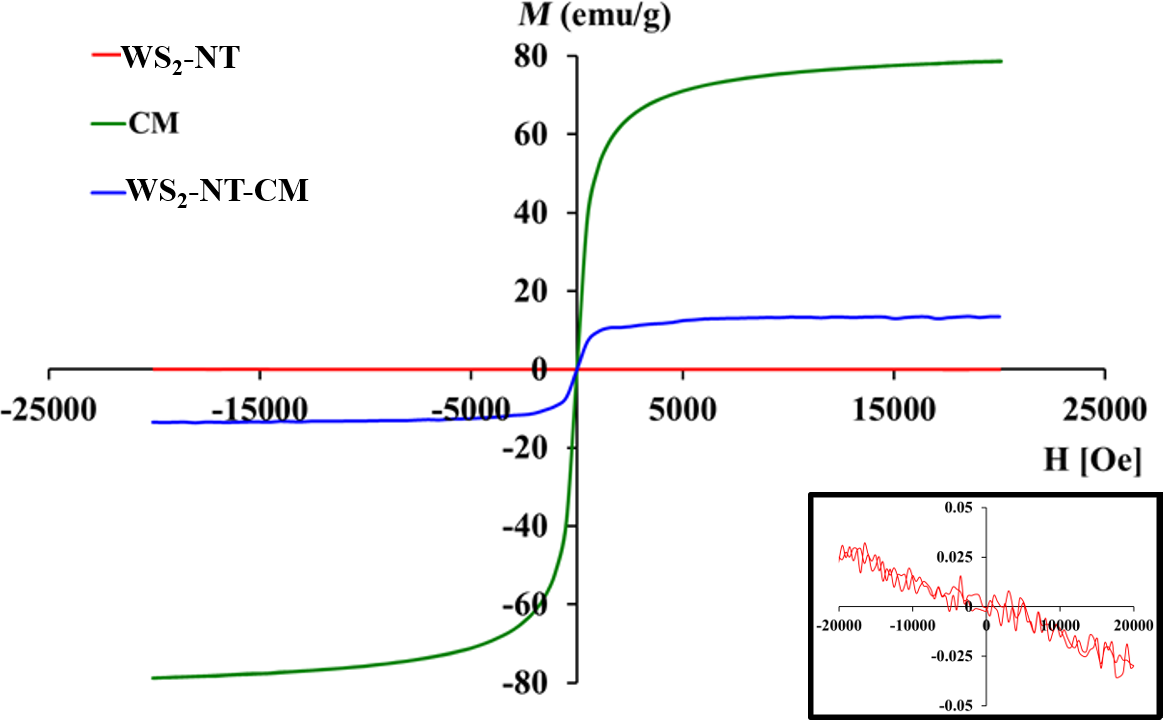
Magnetization spectra of WS_2_-NTs, CAN-mag, and WS_2_-NT-CM, taken at 10 K. Inset is the magnetization spectra of WS_2_-NTs.

Figure 8 shows the magnetization spectra of WS_2_-NT, CAN-mag, and WS_2_-NT-CM nanocomposites, all taken at a temperature of 10 K. For WS_2_-NTs (red spectrum, inset image), the external magnetic field induces a very weak magnetic field in the opposite direction. This means that WS2-NTs are diamagnetic. The CAN-mag curve (green) demonstrates superparamagnetic behavior, where the magnetization increases with the strength of the magnetic field until it approaches saturation, and there is no hysteresis loop. Superparamagnetism is typical for iron-oxide nanoparticles [67]. The nanocomposite WS_2_-NT-CM (blue curve) maintains superparamagnetism, with a saturation value of about ±13 emu/g, which is a sixth of the saturation value for CAN-mag alone. The latter reaches a saturation value of ±78 emu/g, which is consistent with a previous publication [52]. These results are understandable when evaluating the part of CAN-mag in the WS_2_-NT-CM composite. A calculation based on the weight percentages of iron and cerium in the nanocomposite from ICP (Table 1), and the molecular weights of CAN and maghemite, results in 13.2% weight of CAN-mag of the whole composite weight. Taking into account this percentage and the fact that diamagnetism is a weak effect that is always dominated by ferromagnetism (hence, by superparamagnetism), the curve shape and saturation values for WS_2_-NT-CM are to be expected.

When bringing dispersions of CAN-mag nanoparticles and WS_2_-NT-CM close to a magnet, however, it is only the latter that is visibly attracted. The video (Supporting Information File 2) shows the liquid of a WS_2_-NT-CM dispersion gradually clearing up when brought near a magnet, and the composite particles moving towards the magnet. Despite the fact that the magnetization intensity of CAN-mag nanoparticles is higher compared to WS_2_-NT-CM, only the latter is drawn to the magnet, and very slowly, over the course of days. The reason is that CAN-mag nanoparticles are more stable in water than WS_2_-NT-CM (see also the zeta potential results), meaning there are strong electrostatic interactions successfully competing with the magnetic force.

## Conclusion

To summarize, we prepared a nanocomposite of WS_2_-NTs functionalized with CAN-maghemite nanoparticles. The preparation procedures are facile and make use of readily available reagents and equipment. Electron microscopy, FTIR, zeta potential, TGA, and ICP analyses demonstrated the attachment of CAN-mag nanoparticles to the nanotubes. CAN-mag attachment around the nanotubes was not conformal, and in small percentages. Yet, the composite maintained the magnetic character of the nanoparticles. Moreover, the functionalized nanotubes proved to have a higher activity as PTT agents compared to bare WS_2_-NTs in in vitro tests done with HeLa and MCF7 cancer cells.

In addition, functionalization with CAN-mag enabled the attachment of PEI and PAA onto the nanocomposite, as shown by TEM, FTIR, TGA, and zeta potential. The ability for further attachment of polymers and other molecules can be utilized to enhance the therapeutic activity. One way is the attachment of a second PTT agent, such as a polypyrrole, and irradiation in two wavelengths. Another way is the attachment of a photodynamic therapy (PDT) agent. In this preliminary work, we used WS_2_-NTs with a wide size distribution range. A very important step for future research, required prior to in vivo trials, is to use nanotubes in a narrower size range, suitable for targeted PTT.

## Supporting Information

File 1 Selected FTIR characterisation data and viability tests using MCF7 cells.

File 2 A video of a WS_2_-NT-CM dispersion brought near a magnet.
