# Supplementary material for: Tungsten disulfide-based nanocomposites for photothermal therapy
Source: Beilstein J Nanotechnol. 2019 Apr 2;10:811–22. doi: 10.3762/bjnano.10.81 (PMC6466784; doi:10.3762/bjnano.10.81)
Supplement: File 1 — Selected FTIR characterisation data and viability tests using MCF7 cells. [file Beilstein_J_Nanotechnol-10-811-s001.pdf]

BEILSTEIN JOURNAL OF NANOTECHNOLOGY

Supporting Information

for

Tungsten disulfide-based nanocomposites for photothermal therapy

Tzuriel Levin, Hagit Sade, Rina Ben-Shabbat Binyamini, Maayan Pour, Iftach Nachman and Jean-Paul Lellouche

Beilstein J. Nanotechnol. **2019,** *10,* 811–822. doi:10.3762/bjnano.10.81

Selected FTIR characterisation data and viability tests using MCF7 cells

License and Terms: This is a supporting information file under the terms of the Creative Commons Attribution License (http://creativecommons.org/licenses/by/4.0). Please note that the reuse, redistribution and reproduction in particular requires that the authors and source are credited.
The license is subject to the *Beilstein Journal of Nanotechnology* terms and conditions: (https://www.beilstein-journals.org/bjnano)

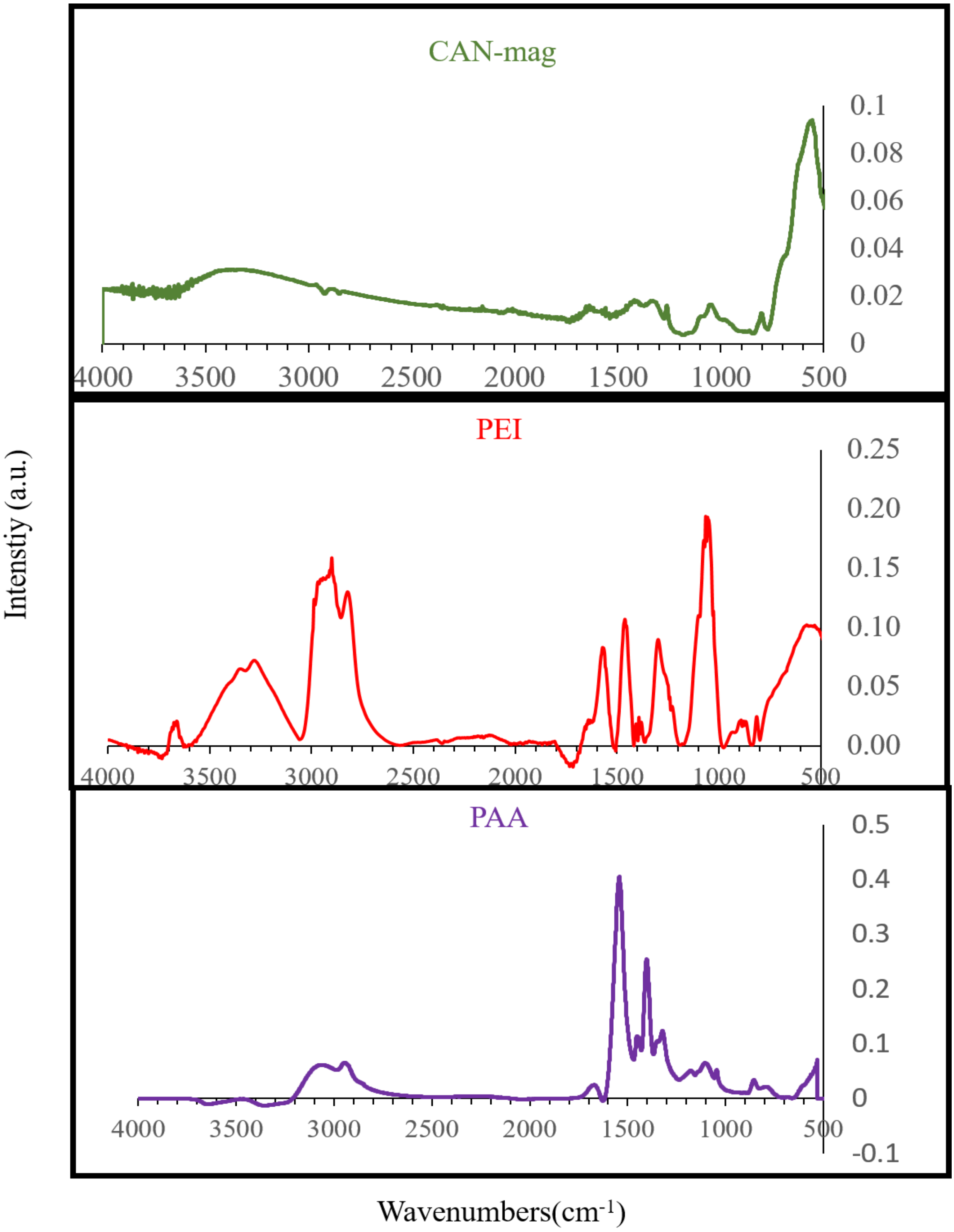

Figure S1: FTIR absorbance spectra of CAN-mag, polyethylenimine, and poly(acrylic acid).

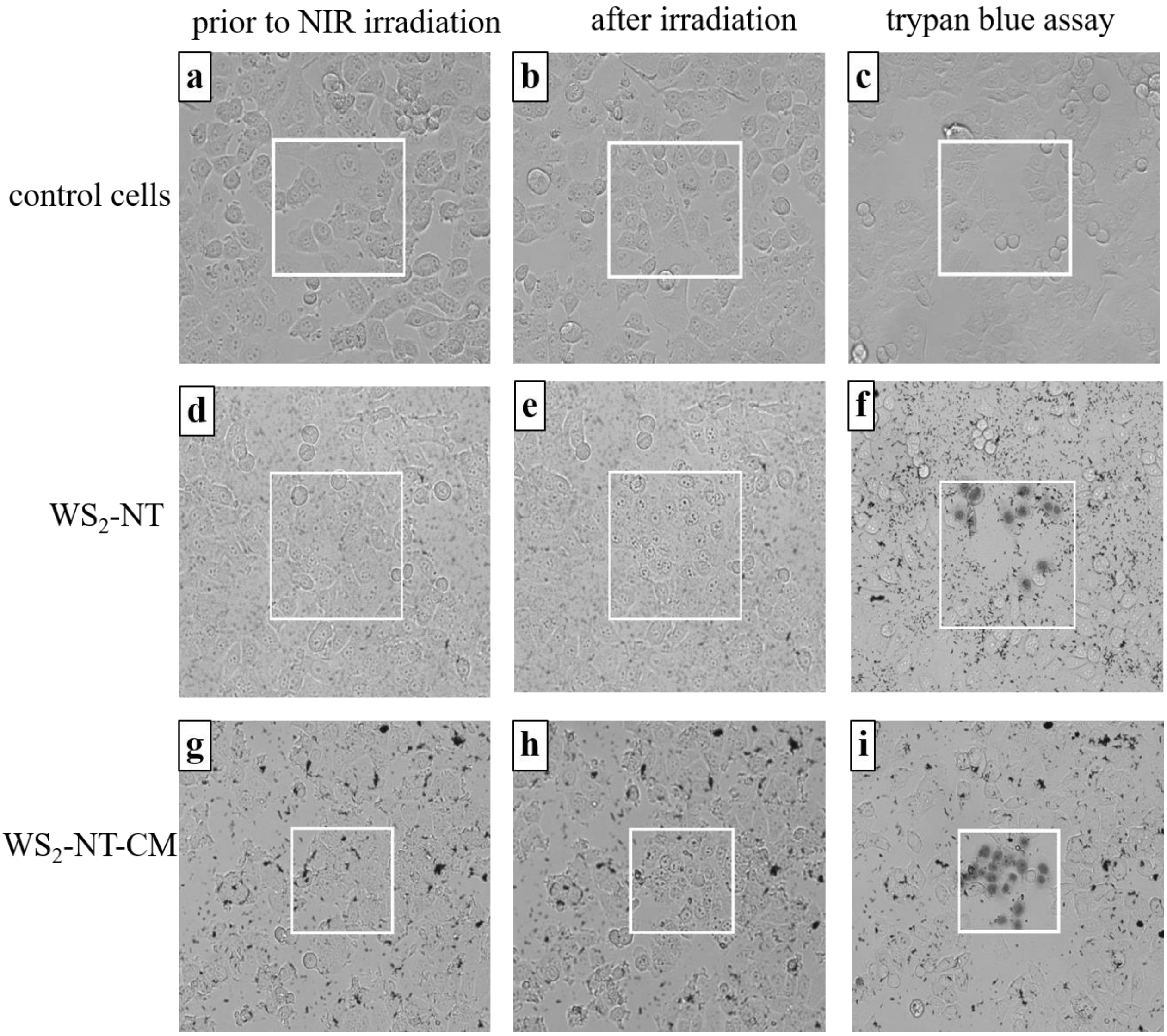

Figure S2: Phase-contrast microscopy images of MCF7 cells. The white squares indicate 157 μm × 157 μm areas irradiated with an NIR (700 nm) laser. Left column: cells prior to NIR irradiation; middle column: after irradiation for 1 minute; right column: after irradiation for 1 minute and application of trypan blue assay. (a–c) Control (untreated) cells; (d–f) Cells pre-incubated with WS_2-NT; (g–i) Cells pre-incubated with WS_2-NT-CM.
